# Subungual Glomus Tumor of the Toe: A Case Report

**DOI:** 10.7759/cureus.104452

**Published:** 2026-02-28

**Authors:** Hala Alrayyes, Noorul Ibtesam Idris, Mohammad Al Bitar, Yusuf Bukhamas, Ahmed Siddiqui

**Affiliations:** 1 Orthopedics, Royal Medical Services, Riffa, BHR; 2 Orthopedics, Royal College of Surgeons in Ireland, Riffa, BHR

**Keywords:** cold exposure, gadolinium-enhanced mri, localized digital pain, subungal glomus tumor, surgical excision

## Abstract

The diagnosis of a rare subungual glomus tumor underscores the importance of maintaining a high index of suspicion among clinicians for patients presenting with unexplained chronic toe pain. Early evaluation with gadolinium-enhanced MRI is essential for the accurate diagnosis of these lesions and timely surgical intervention, which can significantly alleviate prolonged suffering from this condition. This case highlights the often-subtle symptoms and lengthy diagnostic delays associated with glomus tumors, reinforcing the need for heightened clinical awareness in detecting these lesions. Furthermore, surgical excision remains curative, with early intervention minimizing morbidity and achieving favorable patient outcomes. This report advocates for comprehensive preoperative assessments to ensure accurate diagnosis and effective treatment pathways for rare subungual glomus tumors.

## Introduction

Glomus tumors are uncommon benign neoplasms that originate in the glomus apparatus, which is a specialized arteriovenous structure involved in thermoregulation within the dermis [1]. These tumors can develop anywhere in the body. However, they are most commonly found in the subungual region of the fingers [2]. Glomus bodies are less abundant in the toes, making subungual tumors of the toes rare and often underdiagnosed [3].

The clinical presentation of glomus tumors involves the classical triad of paroxysmal pain, localized tenderness, and cold hypersensitivity [4]. Nevertheless, the diagnosis is often delayed due to the tumor’s small size and the subtle clinical presentation, leading to a long history of unexplained pain preceding the diagnosis [5]. Clinical tests to aid the diagnosis include imaging modalities such as ultrasound and MRI, which may initially fail to identify small lesions in many cases [6].

Gadolinium-enhanced MRI is the most sensitive diagnostic tool, typically demonstrating a well-defined, enhancing lesion on T1-weighted and T2-weighted sequences [7]. However, the gold standard for identification and definitive diagnosis remains histopathological examination [8]. Surgical excision is a curative treatment in the majority of cases, providing immediate symptom relief and a low recurrence rate with complete removal of the tumor [9].

We report a case of a 52-year-old woman presenting with a history of chronic distal toe pain for seven years, who was diagnosed with a subungual glomus tumor of the second toe. This case highlights the diagnostic challenges associated with small and atypically located glomus tumors. Additionally, it points out the importance of maintaining a high clinical suspicion in patients presenting with chronic, localized digital pain of unexplained cause.

## Case presentation

A 52-year-old female was referred to our orthopedic surgical clinic on day one with chronic distal toe pain involving the right second toe, persisting for approximately seven years. The pain was described as sharp, localized, and exacerbated by minor trauma, cold exposure, or compression from footwear. The patient denied any preceding injury, infection, discoloration, swelling, numbness, or radiating pain. There were no associated nail deformities, ulceration, or systemic symptoms such as fever or weight loss.

**Figure 1:**
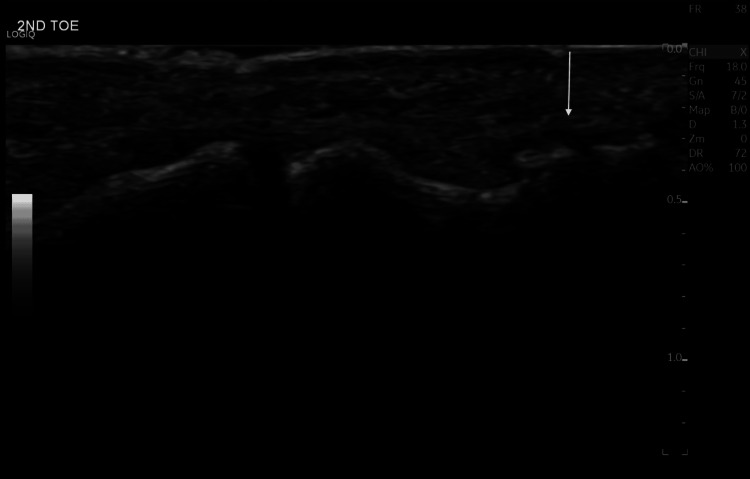
Ultrasound of the second toe of the right foot (dorsal aspect) in 2024. There are no obvious detectable masses, fluid collections, or abnormal flow signals seen in this ultrasound. The interphalangeal joint appears normal and is well-defined, with smooth cortical margins as labeled by the arrow.

**Figure 2:**
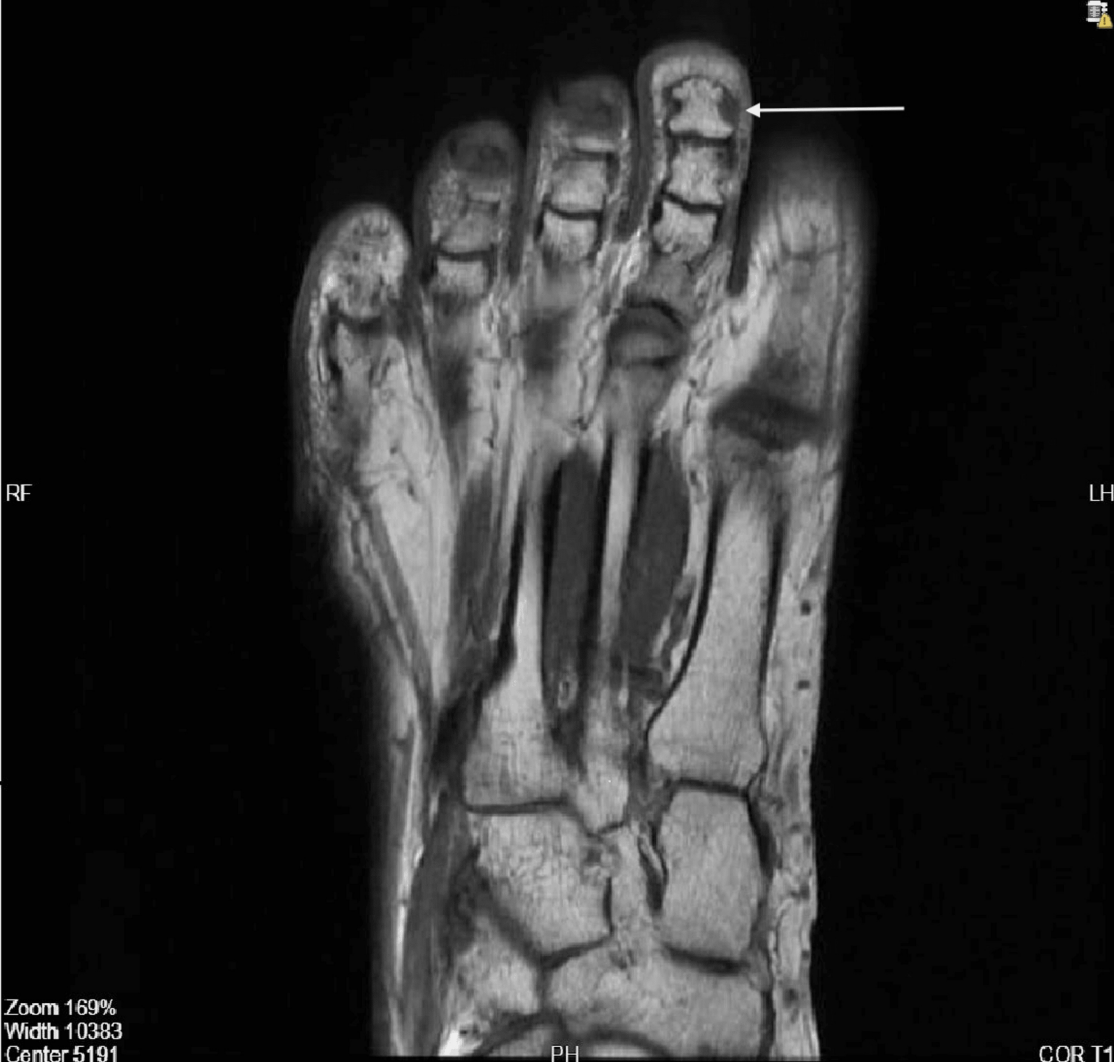
MRI of the second toe of the right foot (2023). There is no evidence of a subcutaneous soft tissue or webspace mass in the second toe of the right foot, as labeled by the arrow.

She had sought multiple consultations during this period, and various imaging studies, including ultrasound and MRI, were performed without a conclusive diagnosis. Earlier ultrasound (2024) evaluations revealed no detectable mass, abnormal flow, or fluid collection, and the interphalangeal joint appeared normal (Figure 1). An MRI performed in 2023 was unremarkable (Figure 2).

**Figure 3:**
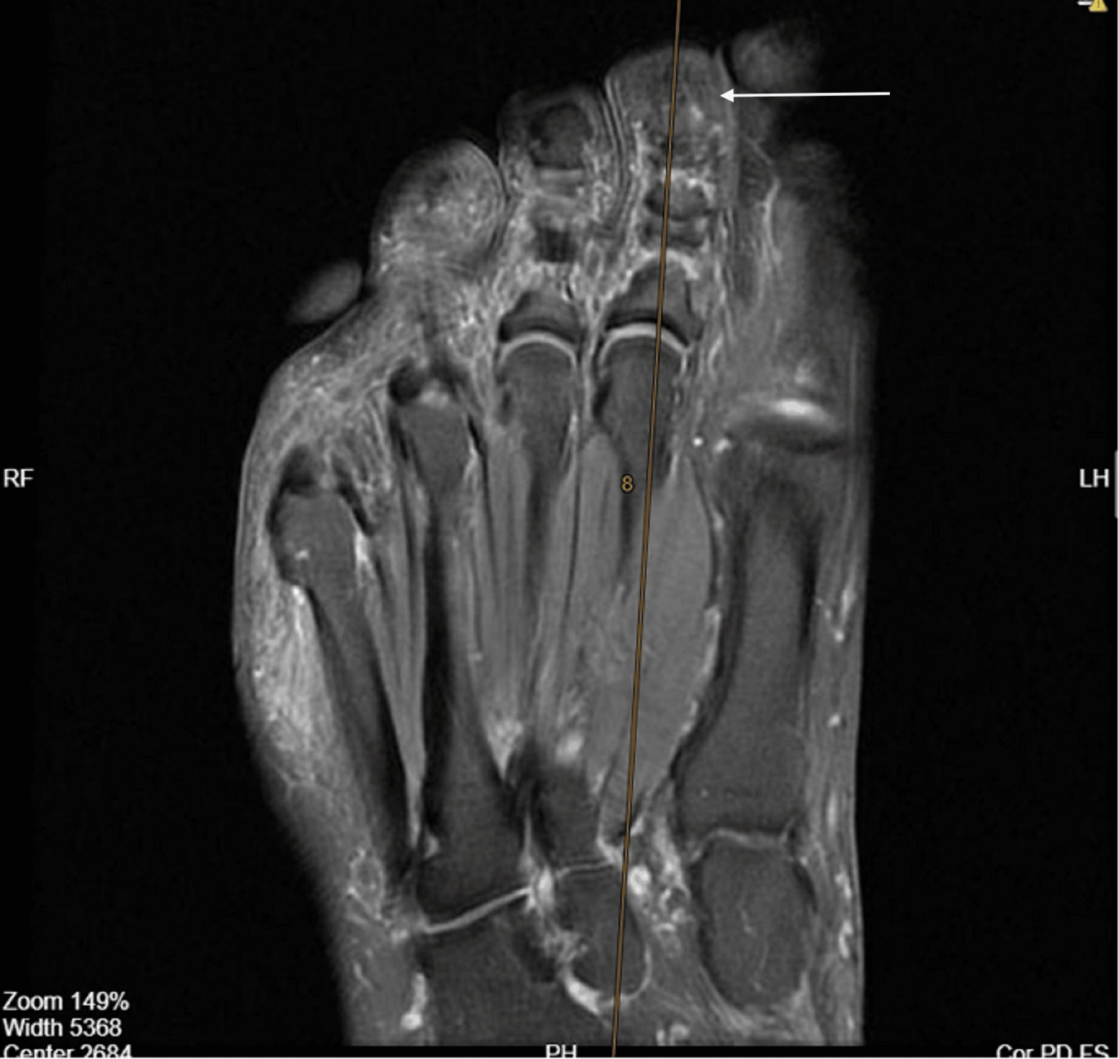
Preoperative MRI of the second toe of the right foot in coronal view (2025). There is evidence of a small, well-defined, rounded, enhancing subcutaneous nodule measuring 2.5x1.5 mm on the dorsal aspect of the tip of the second toe in the right foot, as labeled by the arrow, suggesting a vascular lesion.

On current examination, there was focal tenderness over the distal interphalangeal region without visible swelling, hyperalgesia, or nail deformity. The overlying nail plate was intact, and neurovascular status was preserved. Given the persistent nature of symptoms and localized tenderness, a repeat MRI (1.5T, fat-suppressed T1-weighted with gadolinium) was obtained, revealing a small, well-defined, rounded, enhancing subcutaneous nodule measuring 2.5x1.5 mm on the dorsal aspect of the tip of the second toe, consistent with a vascular lesion such as a glomus tumor or hemangioma (Figure 3).

Under general anesthesia, a longitudinal nail bed incision was performed. The nodule was excised en bloc, including a portion of the nail matrix. Hemostasis was achieved, and the nail bed was meticulously reconstructed. The wound was dressed under strict aseptic technique, and a sterile protective dressing was applied postoperatively.

The patient reported immediate postoperative pain relief and remains symptom-free on follow-up, which is consistent with the successful treatment of a glomus tumor.

## Discussion

Subungual glomus tumors of the toe are infrequently reported, reflecting the relative scarcity of glomus bodies in the distal foot. Our patient experienced prolonged, nonspecific toe pain for approximately seven years, illustrating the well-recognized diagnostic delay in which subtle symptoms are frequently misattributed to trauma, arthritis, or other benign conditions. Subungual glomus tumors are rare, benign vascular neoplasms that typically present with trisymptomatic pain, point tenderness, and temperature-sensitive dysesthesia [4]. Despite these hallmark features, the slow evolution of symptoms is often subtle and commonly attributed to more frequent nail unit disorders, resulting in a protracted diagnostic interval.

MRI, especially with gadolinium enhancement, is the imaging modality of choice for suspected glomus tumors due to its superior sensitivity for tiny intranail lesions and its ability to delineate tumor margins and relationships to surrounding structures [7]. In this case, gadolinium-enhanced MRI detected an occult subungual lesion as small as 2.5x1.5 mm, supporting its role as a highly sensitive diagnostic tool. These imaging features facilitate reliable differentiation from ganglion cysts, epidermoid cysts, and other soft tissue masses. When radiological findings closely correlate with the clinical presentation, MRI plays a pivotal role in confirming the diagnosis, particularly given the low recurrence rates reported following complete surgical excision.

Bedside physical exam tests remain valuable first-line diagnostic tools, particularly when cold sensitivity is a prominent symptom. Hildreth’s test, which involves the application of a proximal tourniquet to induce transient ischemia, may result in temporary pain relief and is considered relatively specific for glomus tumors [10]. Love’s pin test helps localize point tenderness by eliciting focal pain with gentle pressure, while cold-sensitivity testing reproduces characteristic symptoms following exposure to low temperatures [11]. Transillumination may further aid detection by revealing subungual vascular masses that are not readily palpable. Positive findings on these clinical tests raise diagnostic suspicion and support early referral for targeted imaging, thereby shortening the diagnostic interval.

Surgical excision via a longitudinal nail-bed approach remains the definitive treatment for subungual glomus tumors. Complete tumor removal, including an adequate margin of the nail matrix when required, is associated with immediate symptom relief and a low risk of recurrence. Reported recurrence rates of up to 33-50% following incomplete excision underscore the importance of accurate preoperative localization and careful surgical planning to ensure complete tumor clearance [9].

This case reinforces the importance of maintaining a high index of clinical suspicion for glomus tumors in patients presenting with chronic, localized digital pain, even when initial investigations are inconclusive. Early MRI evaluation may shorten the diagnostic pathway, prevent unnecessary investigations, and facilitate timely curative surgery before chronic pain becomes entrenched. Further prospective studies are warranted to evaluate the diagnostic yield of routine MRI in persistent toe pain and to refine referral pathways.

## Conclusions

In summary, this case highlights the significant diagnostic challenges that can accompany subungual glomus tumors, particularly in atypical presentations in the toe. The prolonged history of localized pain experienced by the patient demonstrates the necessity for a high index of clinical suspicion among healthcare providers, especially when standard imaging modalities fail to reveal underlying pathologies.

Gadolinium-enhanced MRI proves to be an invaluable diagnostic tool, effectively identifying small lesions that may otherwise go unnoticed, thus facilitating timely surgical intervention. The successful excision of the tumor not only alleviated the patient’s chronic pain but also underscores the efficacy of surgical management in achieving favorable outcomes for this rare condition. Moving forward, it is imperative that clinicians remain vigilant in recognizing the hallmark symptoms of glomus tumors and consider them in differential diagnoses for patients presenting with unexplained digital pain. Enhanced awareness and early referral for imaging can significantly reduce the diagnostic delay, ultimately leading to more effective and timely treatment strategies.
